# Evaluating footwear “in the wild”: Examining wrap and lace trail shoe closures during trail running

**DOI:** 10.3389/fspor.2022.1076609

**Published:** 2023-01-06

**Authors:** Eric C. Honert, Kathryn Harrison, Daniel Feeney

**Affiliations:** Performance Fit Laboratory, BOA Technology Inc., Denver, CO, United States

**Keywords:** wearable sensors, IMU, plantar pressure, footwear upper, footwear features

## Abstract

Trail running participation has grown over the last two decades. As a result, there have been an increasing number of studies examining the sport. Despite these increases, there is a lack of understanding regarding the effects of footwear on trail running biomechanics in ecologically valid conditions. The purpose of our study was to evaluate how a Wrap vs. Lace closure (on the same shoe) impacts running biomechanics on a trail. Thirty subjects ran a trail loop in each shoe while wearing a global positioning system (GPS) watch, heart rate monitor, inertial measurement units (IMUs), and plantar pressure insoles. The Wrap closure reduced peak foot eversion velocity (measured *via* IMU), which has been associated with fit. The Wrap closure also increased heel contact area, which is also associated with fit. This increase may be associated with the subjective preference for the Wrap. Lastly, runners had a small but significant increase in running speed in the Wrap shoe with no differences in heart rate nor subjective exertion. In total, the Wrap closure fit better than the Lace closure on a variety of terrain. This study demonstrates the feasibility of detecting meaningful biomechanical differences between footwear features in the wild using statistical tools and study design. Evaluating footwear in ecologically valid environments often creates additional variance in the data. This variance should not be treated as noise; instead, it is critical to capture this additional variance and challenges of ecologically valid terrain if we hope to use biomechanics to impact the development of new products.

## Introduction

Trail running participation, particularly in ultra-distances, has grown over the last two decades (1, 2). As a result, recent studies have examined the physiological predictors of trail running performance (3, 4), in-lab biomechanical changes resulting from prolonged downhill runs (5) and trail races (6), and biomechanics and physiology during outdoor trail running (7, 8). Despite this emerging research field, there is a lack of understanding regarding the effects of footwear on trail running. Unfortunately, most of this research is still done in the laboratory, where the unique challenges of trail running are not placed on the participants.

Footwear is a key piece of equipment that that impacts running performance in road running (9, 10). While road running is dominated by sagittal plane motion (11), trail running increases motion in the frontal and transverse planes (7, 11). As such, trail running exacts different demands on footwear than road running. Recent studies have demonstrated that shoe upper material (12) and footwear closure systems (13, 14) can impact biomechanical performance, specifically in non-sagittal plane motions. The upper and fit of footwear is considered a critical design feature by running footwear experts (15), however little research exists on the impact of uppers on trail running performance. While the benefits of alternative shoe upper designs were elucidated in-the-lab during agility-based movements, they have not been evaluated for running “in-the-wild.”

Despite the growing evidence of the validity of wearable technology (16), there is relatively little known about the effects of fit of footwear on the trail in ecologically relevant terrain. Inside of the lab, better fitting shoes have been related to reduced vertical loading rates (*via* force plates) and reduced pronation (eversion) velocities (17). Surrogates of such biomechanical metrics could be captured with wearable technologies such as inertial measurement units (IMUs) that contain accelerometers and gyroscopes. For instance, an IMU attached to the foot can measure foot eversion velocity near foot contact and peak acceleration – a surrogate for vertical loading rate (18). Additionally, IMUs have been used to characterize the terrain of uphill trail running vs. in-lab running through medial-lateral accelerations (7) – with runners exhibiting larger medial-lateral accelerations outdoors. As footwear uppers have been shown to influence agility movement transition times (12, 14) and frontal plane kinematics (13), medial-lateral accelerations may be sensitive to changes in the footwear upper.

Plantar pressure insoles are an additional wearable technology that may be used to evaluate footwear outside-of-the-lab. Plantar pressures have been used to study foot strike patterns on trail [with road shoes (19)], peak and mean pressures during marathons (20), and to quantify differences in pressure distribution during longitudinal training studies (21). Moreover, plantar pressure has revealed differences between lace-up boots and lace-free boots in the field; lace up boots reduce peak pressures under the heel and toes while increasing heel contact area (22). Inside the lab, both increased contact area and reduced peak heel and toe pressures have been shown to differentiate between comfortable orthotics (23) and different footwear features (17, 24). In total, both the heel and the toe region of plantar pressure have been shown to be sensitive to footwear fit and/or subjective comfort.

While evaluating the impact of footwear outside the lab often results in additional variation in the data, this variation is indicative of real-world usage and experiences. In fields such as cognition (25) and neuroscience (26), the effects of interventions can be missed if only tested in the laboratory due to a lack of context or ecological validity. While testing running biomechanics on a treadmill provides many observations in a tightly controlled setting, assessments of footwear on a treadmill may not generalize to overground running (27, 28) and will certainly not represent the challenges of the terrain where subjects will use trail footwear. As a result, using a treadmill to test trail running footwear limits the generalizability of findings and may be entirely inappropriate to assess this type of footwear. This limitation extends to other commonly tested biomechanical interventions such as prostheses, exoskeletons, and road running footwear. If we hope to bridge the gap between biomechanics and product design, researchers must expand their testing toolbox to include wearable sensors, explore the critical variation that will be present in the data, and design experiments in such a way to rigorously test product in ecologically relevant terrain.

The purpose of our study was twofold: (1) to evaluate how changing a trail shoe closure system impacts trail running biomechanics and performance, and (2) to highlight an experimental paradigm for testing biomechanical interventions on a trail. Specifically, we evaluated trail running in the La Sportiva Cyklon with a Wrap closure and a Lace closure with respect to preference and performance. We hypothesized that the Wrap closure would provide a better fit than the Lace closure. We anticipated a shoe with a better closure would: (1) reduce foot eversion velocity, (2) reduce loading rate as measured by peak acceleration or peak jerk, (3) reduce the medial/lateral acceleration, (4) reduce peak pressure in the toe and/or the heel, and (5) increase the contact area in the toe and/or the heel. We evaluated the peak jerk in addition to the peak acceleration as jerk provides a similar time-derivative as loading rate. We also evaluated subjective and objective (heart rate) measures of exertion to ensure that there was no difference in effort between the two shoes.

## Materials and methods

### Participants

**Figure 1:**
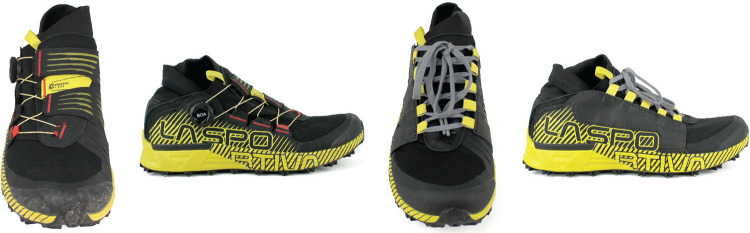
The La sportiva cyklon with the wrap upper (left) and the retro-fitted lace version of the shoe (right).

Thirty subjects (15 male, 15 female means ± standard deviations: height: 172 ± 6 cm, mass: 65 ± 8 kg, age: 34 ± 8 yrs) provided written informed consent prior to participation in trail running. The protocol was approved by the Institutional Review Board (protocol: 22-BOAT-101). Participants with US men's shoe size 10 and 11 and US women's shoe size 7.5 and 9 were recruited for this study. To be eligible to participate, subjects were required to run at least 24 km per week and participate in trail running once per week (weather permitting) with no injuries that resulted in missing more than a week of running in the past six months. All participants ran in the La Sportiva Cyklon (Wrap) and a retrofitted La Sportiva Cyklon with Laces (Lace) (Figure 1).

### Protocol

**Figure 2:**
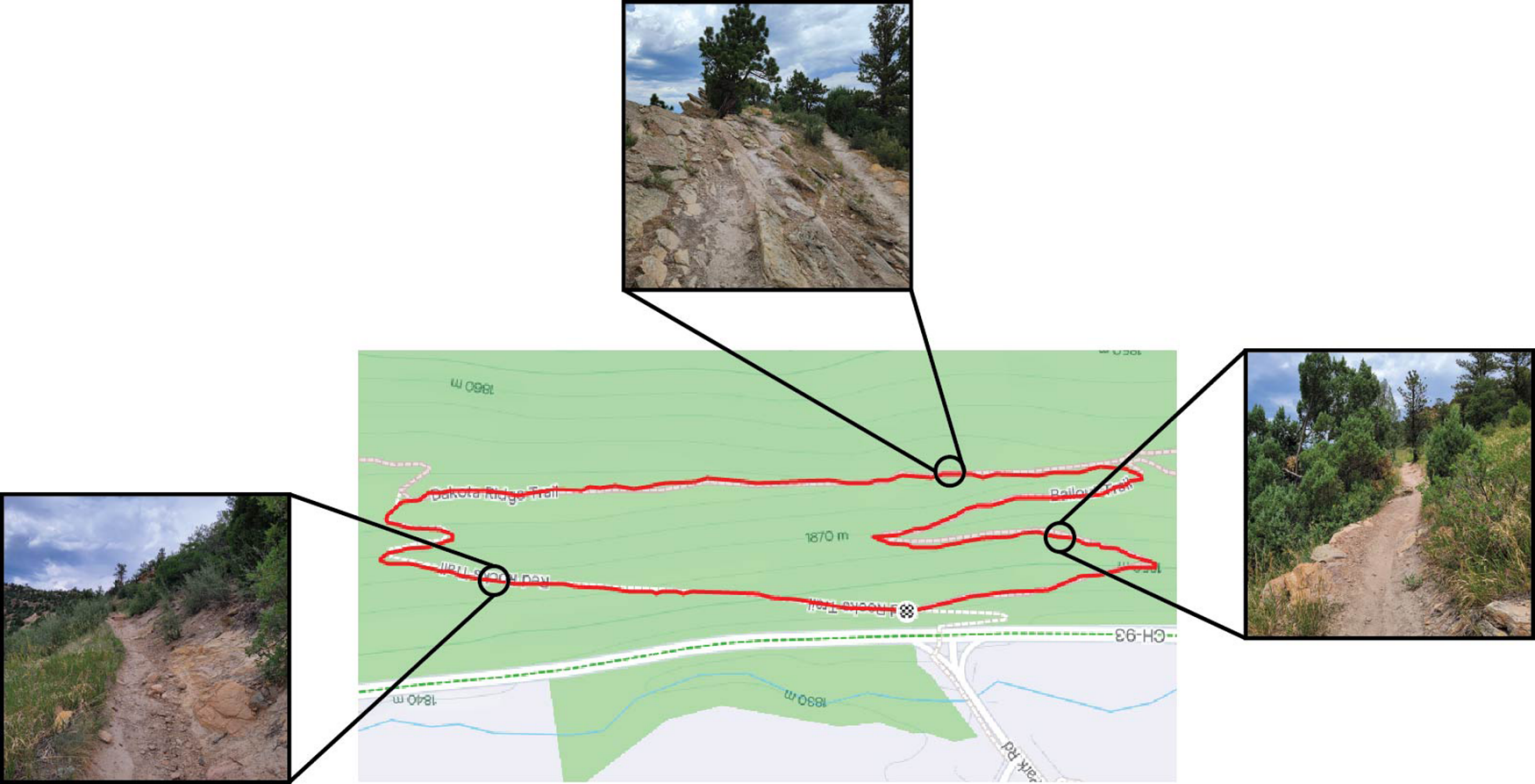
Topographic map with route subjects ran (shown in red). Subjects ran the route in a counter-clockwise fashion. Pictures show example terrain of the different trail sections: uphill (right), top (top), and downhill (left). The average uphill slope was +4°, the average top slope was +2°, and the average downhill slope was −6°. Topographic map was used with permissions from Strava (http://www.strava.com).

The trail run was performed on a 1.6 km loop near Morrison, Colorado, USA (Figure 2) and had three sections. The uphill portion of the trail was approximately 580 m and had an average incline of four degrees (varied between 13 degrees uphill and one degree downhill). The top portion of the trail was more technical than the other two sections. This portion of the trail was approximately 520 m with an average incline of two degrees (varied between nine degrees uphill and two degrees downhill). The downhill portion of the trail was approximately 470 m with an average decline of six degrees (varied between three and 11 degrees downhill). The technicity of the different portions of the trail, according to the international mountain bicycling association, were intermediate (blue) for the uphill and downhill and advanced (black) for the top. The participants first ran a warm-up loop with a trained experimenter to become familiar with the terrain and the loop. The subjects then ran the loop another four times to test the shoes in a randomized counterbalanced order (A-B-B-A) to account for familiarization and fatigue. Subjects were encouraged to run at similar speeds between each lap. Data were collected from IMUs (±16 g and ±2,000°/sec at 1125 Hz with 16-bit sensitivity, ±200 g at 1,600 Hz with 13-bit sensitivity, IMeasureU, Denver, USA) attached to the heel counter, plantar pressure sensors (100 Hz, XSENSOR, Calgary, CAN), a global positioning system (GPS) watch (1 Hz, Suunto, Vantaa, Finland), and an optical heart rate sensor (1 Hz, Polar Verity Sense, Polar Electro Inc., Kempele, Finland) attached to the upper arm. Previous versions of this heart rate sensor have been validated against electrocardiograms during level and uphill running (29). GPS coordinates and heart rate data were synchronously recorded on the GPS watch. If subjects had a greater than one minute difference between their laps, their data were excluded from further analyses. Prior to each lap, subjects performed three synchronizing jumps to indicate when the subjects began running. After these jumps, the subject started the GPS watch and began running.

### Subjective outcomes

After running the warm-up lap, the subjects reviewed the questionnaire. After each lap in a study condition, subjects rated their exertion on an ordinal scale of 0 (not tired) to 10 (exhausted). After the second run in each shoe, they responded to questions regarded the performance of the shoe for uphill, level, and downhill running. Subjects also rated their confidence while running in the shoe. Finally, subjects rated the overall fit, the forefoot fit, midfoot fit, and the heel fit. All questions besides the fit for the specific foot regions were rated on an ordinal scale from zero to 10, where zero was “poor” and 10 was “great”. For the specific foot regions (e.g., forefoot), zero corresponded with too loose, five was perfect and 10 was too tight. Similar ordinal scales have been shown to be a reliable assessment of footwear comfort during running (30). For the full questionnaire see **Supplementary material**.

### Biomechanical outcomes

The IMUs (three-axis accelerometer, three-axis gyroscope) were used to determine running speed, peak acceleration (sometimes referred to as shock), jerk, medial-lateral acceleration ranges, and peak eversion angular velocity. We first combined the low-g and high-g accelerometer data to avoid saturation that can occur during running (31). This was accomplished by interpolating the high-g accelerometer (1,600 Hz) to the low-g accelerometer frequency (1,125 Hz) with the UNIX timestamps that accompanied each sample. The high-g accelerometer datapoints were then substituted for the low-g datapoints whenever the low-g accelerometer was saturated (at ±16 g). Running strides were then segmented based on the magnitude of the jerk squared (32). Next, both the accelerometer and gyroscope signals were bypass-filtered with a second-order, low-pass 50 Hz Butterworth filter. Using these filtered signals, the IMU-based running speed was determined based on previously published algorithms (32) that have been evaluated for different speeds and slopes of running (33). In short, we computed stride length and divided it by stride time, as defined by subsequent foot contact detections. Stride length was determined through double integrating the acceleration once it had been corrected for gravity. We utilized linear de-drifting to correct for integration error based on zero velocity updates at mid-stance (32, 34). Based on the filtered accelerometer signals, peak acceleration magnitude, peak jerk magnitude, and the medial/lateral accelerometer range [similar to (7)] over the stride were determined. For the peak eversion angular velocity, we filtered the original gyroscope signal with a second-order, bi-pass, low-pass 30 Hz Butterworth filter to better capture running kinematic frequencies and to limit the effects of transient peaks in the gyroscope signal near foot contact. The peak eversion angular velocity was then computed for the first 20% of the stride to capture the foot contact portion of the stride. All IMU metrics were extracted after the detected three hops for the entire running loop.

Average heel and toe contact area along with peak toe and peak heel pressures during the stance phase of running were extracted from the plantar pressure. Running stance phase for the plantar pressure was detected with implementing a gradient decent algorithm on the total insole force to provide foot-contact and toe-off events. The heel region was defined as the rear 20% of the plantar pressure sensor. The toe region was defined as the front 20% of the sensor. These two regions are defined though the XSENSOR software and approximately correspond to the respective regions of the anatomical foot. All pressure metrics were extracted after the detected three hops for the entire running loop.

The GPS coordinates from the watch were used to segment the trail into three portions: uphill, top technical, and downhill (see Figure 2 above) based on the latitude and longitude. As the GPS watch was started immediately after the three synchronizing hops, the biomechanical metrics were examined for each section separately. Average heart rate for each section was computed for each section to evaluate physiological exertion. All biomechanical data processing was performed in Python version 3.9.7.

### Statistics

Linear mixed effects models were used to evaluate the statistical difference between the Wrap and Lace shoes for the subjective outcomes along with biomechanical outcomes. All statistical analyses were performed in R Statistical Software (version 4.1.2) with the LMER (35) and emmeans (36) packages. We first created a linear mixed effects model to evaluate the difference in subjective responses between the configurations (Config: Lace or Wrap) and average heart rate from each trail section that utilized an independent (random) intercept for each subject (Equation 1). Differences in average heart rate were based on estimated marginal means.


(1) Outcome∼Config+(1|Subject)

All other biomechanical outcomes (“*Outcome*”) were then evaluated with a mixed effects model with an independent (random) intercept for each subject and independent slope encoding the subject-specific effect of switching from one shoe condition to the other (Equation 2). In this model, the biomechanical outcomes (e.g., peak eversion velocity) from each detected stride (IMU) or step (plantar pressure) from each subject were utilized. Statistical differences for the biomechanical outcomes were evaluated separately for each of the different trail sections (i.e., uphill, top, downhill) as the biomechanical outcomes are speed dependent. For all statistical tests, *α* was set to 0.05. Presented percent differences were with respect to estimated marginal means. These models use a similar structure to recent work from our lab (13, 14) and represent a natural way to model the subject-specific responses to footwear (37).


(2) Outcome∼Config+(Config|Subject)

## Results

One subject was excluded from analyses because their trail running laps had a range greater than one minute (2 min. 23 s).

### Subjective outcomes

The Wrap shoe was subjectively rated better for uphill, level, and downhill running (*p* ≤ 0.002, Table 1). The Wrap shoe provided subjectively more confidence than the Lace shoe (*p* < 0.001). Though the Wrap shoe was rated with a better overall fit (*p* < 0.001), the Wrap shoe was rated as tighter than the Lace shoe in the forefoot (*p* = 0.04) with no differences in rating for the midfoot or heel fit (*p* > 0.1).

**Table 1:** Subjective feedback regarding the performance of the shoes on different terrain and the fit of the shoes. Presented are study means ± standard deviations (*N* = 29).

Configuration	Uphill	Level	Downhill	Confidence	Overall Fit	Forefoot Fit	Midfoot Fit	Heel Fit
Wrap	8.3 ± 1.1	8.4 ± 1.2	8.4 ± 1.5	8.8 ± 1	8 ± 1.5	6.2 ± 1.5	5.3 ± 0.6	5.1 ± 0.7
Lace	7.3 ± 1.1	7.5 ± 1.3	6.3 ± 1.5	6.8 ± 1.5	6.5 ± 1.4	5.5 ± 2.1	5.1 ± 1.5	4.5 ± 1.4
*p*-value	<0.001	0.002	<0.001	<0.001	<0.001	0.04	0.7	0.1

### Exertion outcomes

There were no statistical differences in either subjective (*p *= 0.46) or objective exertion outcomes (*p* > 0.17, Table 2). The average heart rate difference between Wrap and Lace shoes was less than one beat per minute on all sections of the trail (Table 2).

**Table 2:** Biomechanical metrics examined during the trail running loop. The bolded metrics indicate that there were significant differences between the Wrap and the Lace for each of the different trail sections. Presented are study means ± standard deviations (*N* = 29).

Metric	Uphill		Top		Downhill	
	Wrap	Lace	Wrap	Lace	Wrap	Lace
Heart Rate [BPM]	160 ± 11	159 ± 11	171 ± 11	171 ± 11	162 ± 11	161 ± 11
**Running Speed [m/s]**	2.71 ± 0.38	2.68 ± 0.38	2.54 ± 0.38	2.51 ± 0.38	3.32 ± 0.54	3.32 ± 0.54
Peak Jerk [m/s^3^]	24,660 ± 5202	24657 ± 5891	27,836 ± 5310	27,912 ± 614	40,343 ± 10,490	39,819 ± 11,207
Peak Acc. [m/s^2^]	124.7 ± 21.0	124.1 ± 21.5	135.9 ± 20.5	135.4 ± 22.1	185.9 ± 36.6	182 ± 35
**Peak Ev. Vel. [°/s]**	328.1 ± 77.0	343.8 ± 81.9	324.6 ± 71.6	341.2 ± 75.9	466.7 ± 121.2	490.2 ± 118.5
M/L Acc. Range [m/s^2^]	93.0 ± 22.6	95.2 ± 25.8	105.0 ± 19.4	107.2 ± 22.6	165 ± 40.4	167.4 ± 43.6
**Heel Contact [%]**	61.7 ± 10.8	59.3 ± 11.3	62.8 ± 8.6	60.5 ± 9.7	67.4 ± 5.9	65.4 ± 6.5
Peak Heel Press. [kPa]	180.1 ± 69.5	175.5 ± 65.7	207.1 ± 65.7	205.4 ± 68.4	382.3 ± 87.2	372.9 ± 91.5
Toe Contact [%]	70 ± 5.4	69.8 ± 4.8	70.9 ± 5.9	70.7 ± 5.4	69.2 ± 5.4	69 ± 5.4
Peak Toe Press. [kPa]	513.1 ± 102.9	509.7 ± 105	527.3 ± 100.2	523.9 ± 96.4	511 ± 86.7	516.8 ± 88.9

Abbreviations: Acc., Acceleration; Ev. Vel., Eversion Velocity; M/L, medial-lateral; Press., pressure.

### IMU outcomes

**Figure 3:**
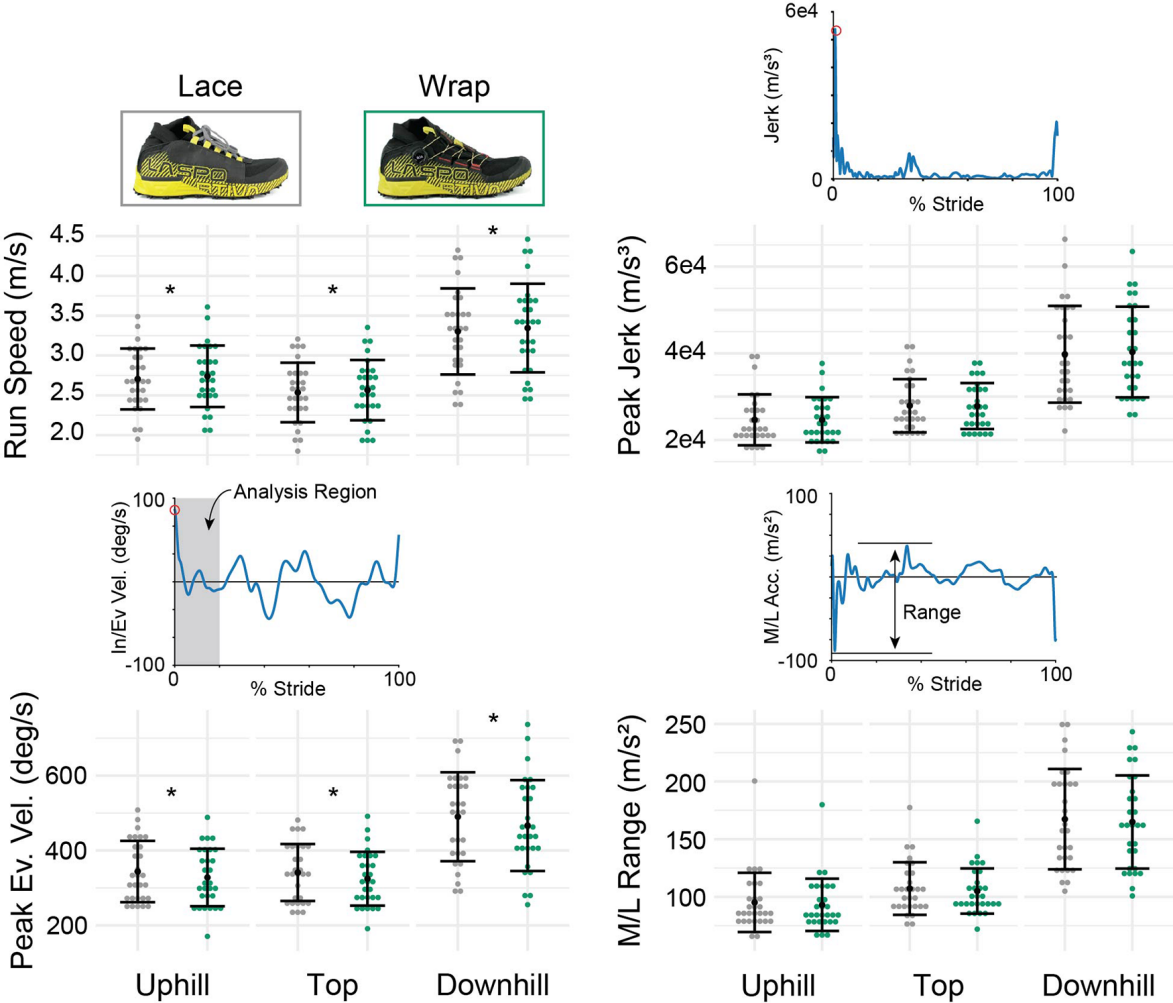
IMU-based outcomes. The individual dots represent subject-average results. The black dots and adjoining error bars represent study averages and standard deviations (*N* = 29). Asterisks (*) indicate significant differences between the Lace and the Wrap shoes for each of the different sections of the trail. Above the peak jerk, peak eversion velocity (Ev. Vel.), and medial/lateral acceleration range (M/L Range) are example curves from one subject and one stride to demonstrate how the metric was computed.

Subjects ran slightly faster in the Wrap than in the Lace shoe on all three sections of the trail (average difference: 0.03 to 0.05 m/s, *p* < 0.022, Figure 3, Table 2). Peak jerk, peak acceleration, and the medial/lateral acceleration range were not significantly different between the two shoes for any of the trail sections (*p* > 0.35, *p* > 0.08, and *p* > 0.20, respectively). The peak eversion velocity was on average 5% lower in the Wrap than the Lace for the uphill (*p *= 0.025), technical top (*p *= 0.008), and downhill (*p *= 0.043) portions of the trail.

### Pressure outcomes

**Figure 4:**
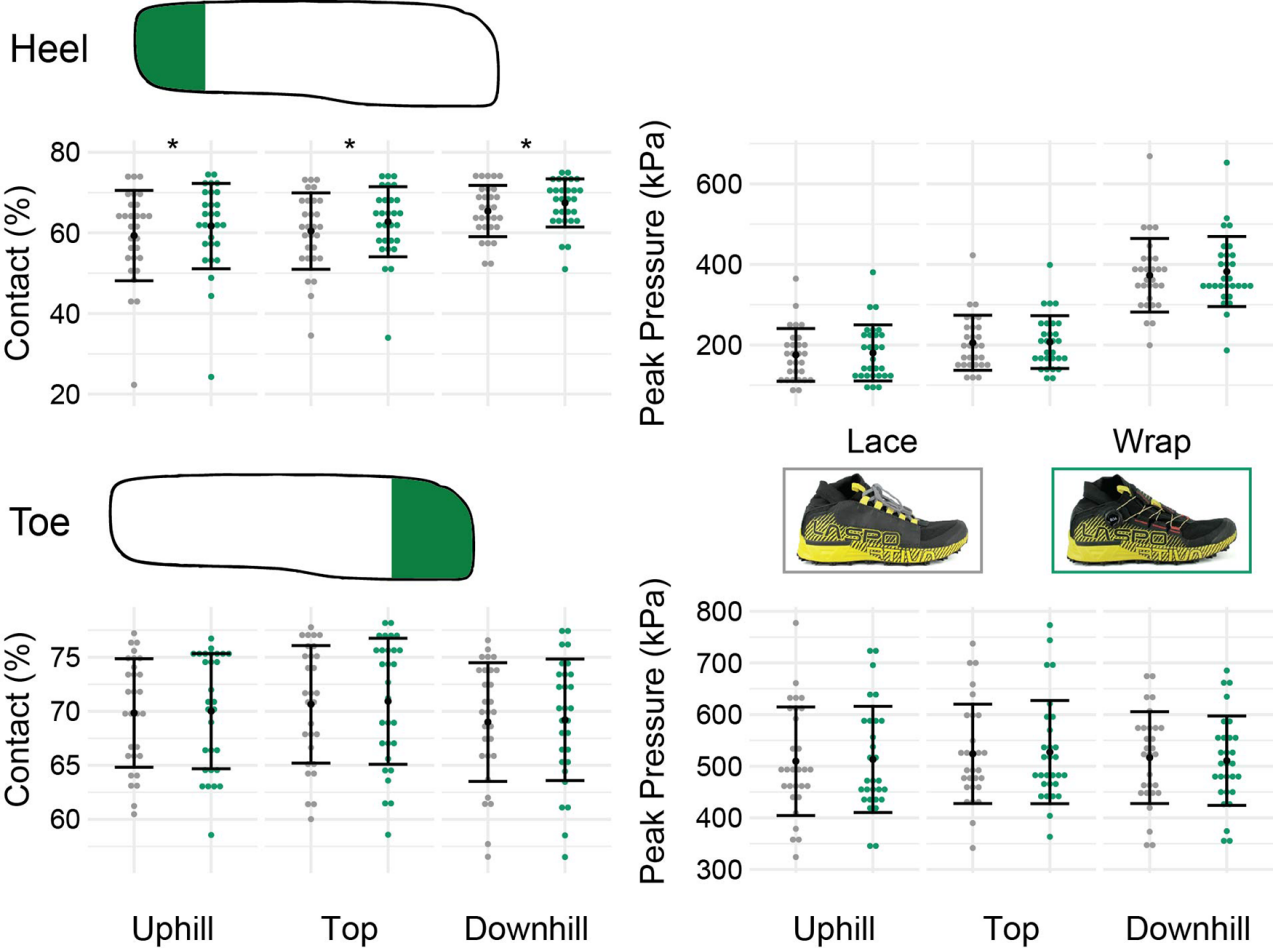
Plantar pressure outcomes. The top row are heel outcomes with a depiction of the heel region in the XSENSOR insoles. The bottom rows are toe outcomes with a depiction of the toe region in the XSENSOR insoles. The individual dots represent subject-average results. The black dots and adjoining error bars represent study averages and standard deviations (*N* = 29). Asterisks (*) indicate significant differences between the lace and the wrap shoes for each of the different sections of the trail.

The average percent heel contact area during the stance phase of running increased by an average magnitude of 2% in the Wrap over the Lace for all sections of the trail (*p* < 0.001, Figure 4, Table 2). The average toe contact area during the stance phase of running was not different between the two shoes for any section of the trail (*p *> 0.21). Both the peak heel and toe pressures did not differ between the two shoes for any section of the trail (*p *> 0.087).

## Discussion

This was the first study to evaluate trail-specific footwear on ecologically valid terrain. We observed a statistically significant decrease in peak eversion velocity, slightly faster running speed, and increase in heel contact area in the Wrap shoe with no differences in qualitative or quantitative measures of exertion. These biomechanical changes were accompanied by greater qualitative scores in overall fit and confidence while running on each part of the trail. Similarly, various BOA wrap configurations have reduced frontal plane kinematics and times to change direction during agility movements and were accompanied by increased qualitative fit (13). In contrast to two of our hypotheses, there were no differences in peak accelerations or medial-lateral accelerations in the Wrap Shoe. We also did not observe any differences in the toe contact area, peak toe pressure, nor the peak heel pressure. Given the observed differences in footwear conditions, we propose the counterbalanced testing of product on a trail with wearable sensors is a valid design that should be used for trail product and may be extended to evaluate other biomechanical interventions.

### Biomechanical features of fit

The Wrap shoe exhibited biomechanical features of a better fitting shoe along with subjects preferring the overall fit over the lace. We observed a significant decrease in peak eversion velocity in the Wrap shoe relative to Lace on each segment of the trail. Similar effects have been observed in better fitting footwear (17). Such a reduction may indicate that there is a better coupling between the footwear and the shoe. In fact, several subjects anecdotally mentioned that there was more relative motion (or slop) in the Lace condition. Future studies may want to examine the connection between this frontal plane velocity and the risk of ankle sprains. Medial and lateral ankle sprains result from excessive eversion/inversion angular velocities and angles (39, 40) and ankle sprains are the most common injury amongst trail runners (38).

We did not observe a difference between the Wrap and Lace in surrogate measures for vertical loading rate, which has been associated with footwear fit (17). Peak acceleration is proposed as a wearable-based surrogate for vertical loading rate (18), we also investigated peak jerk as it provides the same time-derivative as vertical loading rate. The acceleration and jerk magnitudes were dominated by the vertical and anterior-posterior directions, which could indicate these measurements are more sensitive to sagittal plane fit. As we did not observe a difference in the peak jerk nor the peak acceleration magnitudes, but we did observe a difference in the peak eversion velocity: the Wrap shoe has greater implications for footwear fit in the frontal plane rotation rather than the sagittal plane.

In addition to examining biomechanical metrics related to fit, we explored the relations between subjective feedback and metrics from wearables. Such connections between the perception of fit and biomechanics have been previously established using plantar pressure (23). We observed a significant increase in the heel contact area in the Wrap shoe which may contribute to the improvement in the “Downhill Performance” score (Table 1). Over a third of the subjects reported (in the free form response, see Supplementary Material) that they were sliding forward within the Lace shoe during downhill running which could result in less heel contact area. However, the heel contact area may have multiple implications as the Wrap had improved heel contact area on each of the different sections of the trail. We speculate that while the foot may slide forward within the shoe during downhill running, it may slip in the vertical direction during uphill running as runners tend to transition towards a midfoot strike pattern (19), challenging the ability of the upper to hold the heel in contact with the midsole. Seven subjects stated that the Lace had more heel slipping, which could implicate this metric as a quantitative estimate for heel hold. In the future, new technologies may be needed to better connect subjective feedback regarding footwear sliding with biomechanical outcomes such as plantar pressure that can measure shear [which has thus far only been prototyped (41)].

### The debate: Highly controlled vs. ecologically valid

There are trade-offs between performing tightly controlled experiments within the lab compared with more variable data obtained in ecologically valid environments. Mainly, there is greater variability on the trail vs. in the lab. This variability can manifest from changes in temperature, trail conditions, subject foot placement, wind, and more. While laboratory settings tightly control these variables, trail running shoes need to perform in such conditions and therefore need to be tested as such. The variation in data collection conditions increases the variability in biomechanical outcomes, resulting in the need to recruit enough subjects and collect enough observations per subject (e.g., steps) to maintain statistical power. A mixed effects model that includes each individual step from each subject allows for the inter-individual variability in biomechanical measures to be accounted for in the analysis. Critically, footwear features such as fit and outsole features are often designed to mitigate the exact challenges that contribute to this variability such as sloppy rocks and steep gradients. Therefore, to test these design features it is essential to test these products on a trail. To the second aim of our study, we performed a repeatable experimental design to test trail products in an ecologically valid setting while limiting confounding variables. We segmented the trail into three unique sections *via* GPS (uphill, flat, and downhill) to be included as a covariate, instructed all participants to run at similar speeds for each section of a trail, and used a trail loop that could be completed four times within a single session without substantial changes in RPE so subjects could be tested in a counterbalanced order. We also measured their running speed, heart rate, and rating of perceived exertion, which could be included as covariates in our statistical model. For instance, including running speed (measured *via* IMU, Figure 3) within a statistical model (Equation 3) did not change the outcomes of the study; however, it did reduce the *p*-values for each of the IMU outcome significant findings.


(3) Outcome∼Config+Speed+(Config|Subject)

Trail terrain is a critical benefit to evaluating the effects of footwear features. In the laboratory, it is common to evaluate shoe features [e.g., (42, 43)] and biomechanical adaptations (44) on an instrumented treadmill. Treadmill running instigates unique biomechanics [as compared to overground (27),], which can be altered with changing the treadmill belt surface (45) or technicity (11). Such unique adaptations can influence footwear feature biomechanical effects (28). Furthermore, transitioning from the treadmill to the trail induces unique biomechanical adaptations (7). As the footwear we studied here are intended to be used on trail, we propose that these shoes must be tested with the specific biomechanical adaptations that are instigated by trail running. Though we observed difference between the lace and wrap upper configurations on trail, it is an open question as to if footwear interventions instigate similar biomechanical effects indoors vs. outdoors.

### Limitations

There are several limitations to acknowledge. Both the experimenters and the subjects were unable to be blinded to the footwear conditions. Additionally, the insole and IMU data were not able to be aligned post-hoc. As such, statistical analyses that account for running speed (Equation 3) could not be performed with the plantar pressure outcomes.

## Conclusion

The wrap shoe provided improved qualitative and quantitative fit as compared to the lace version of the same shoe. Moving forward, shoe manufacturers and trail runner alike may want to choose shoes with wraps over laces for enhanced fit.

## Data Availability

The raw data supporting the conclusions of this article will be made available by the authors, without undue reservation.
