# Supplementary material for: Evaluating footwear “in the wild”: Examining wrap and lace trail shoe closures during trail running
Source: Front Sports Act Living. 2023 Jan 6;4:1076609. doi: 10.3389/fspor.2022.1076609 (PMC9853429; doi:10.3389/fspor.2022.1076609)

These two shoes are exactly the same besides the upper. As you can see, one pair of shoes has laces while the other has a BOA fit system. During the upcoming running laps, I want you to think about how these shoes affect your running and the comfort of the shoes.

Shoe A1. What was your exertion during this run?

| Not Tired | | |  |  |  |  |  |  |  | Exhausted |
| --- | --- | --- | --- | --- | --- | --- | --- | --- | --- | --- |
| 0 | 1 | 2 | 3 | 4 | 5 | 6 | 7 | 8 | 9 | 10 |

Shoe B1. What was your exertion during this run?

| Not Tired | | |  |  |  |  |  |  |  | Exhausted |
| --- | --- | --- | --- | --- | --- | --- | --- | --- | --- | --- |
| 0 | 1 | 2 | 3 | 4 | 5 | 6 | 7 | 8 | 9 | 10 |

Shoe B2. What was your exertion during this run?

| Not Tired | | |  |  |  |  |  |  |  | Exhausted |
| --- | --- | --- | --- | --- | --- | --- | --- | --- | --- | --- |
| 0 | 1 | 2 | 3 | 4 | 5 | 6 | 7 | 8 | 9 | 10 |

Shoe A2. What was your exertion during this run?

| Not Tired | | |  |  |  |  |  |  |  | Exhausted |
| --- | --- | --- | --- | --- | --- | --- | --- | --- | --- | --- |
| 0 | 1 | 2 | 3 | 4 | 5 | 6 | 7 | 8 | 9 | 10 |

Shoe B2.

How do you feel this shoe performed running uphill?

| Poor |  |  |  |  |  |  |  |  |  | Great |
| --- | --- | --- | --- | --- | --- | --- | --- | --- | --- | --- |
| 0 | 1 | 2 | 3 | 4 | 5 | 6 | 7 | 8 | 9 | 10 |

How do you feel this shoe performed running level ground?

| Poor |  |  |  |  |  |  |  |  |  | Great |
| --- | --- | --- | --- | --- | --- | --- | --- | --- | --- | --- |
| 0 | 1 | 2 | 3 | 4 | 5 | 6 | 7 | 8 | 9 | 10 |

How do you feel this shoe performed running downhill?

| Poor |  |  |  |  |  |  |  |  |  | Great |
| --- | --- | --- | --- | --- | --- | --- | --- | --- | --- | --- |
| 0 | 1 | 2 | 3 | 4 | 5 | 6 | 7 | 8 | 9 | 10 |

What was your confidence level while running in this shoe (e.g. running downhill, technical terrain)?

| Timid | |  |  |  |  |  |  |  | Confident | |
| --- | --- | --- | --- | --- | --- | --- | --- | --- | --- | --- |
| 0 | 1 | 2 | 3 | 4 | 5 | 6 | 7 | 8 | 9 | 10 |

How was the overall fit of the shoe?

| Uncomfortable | | | |  | |  | |  | |  | |  |  | | Comfortable | | |
| --- | --- | --- | --- | --- | --- | --- | --- | --- | --- | --- | --- | --- | --- | --- | --- | --- | --- |
| 0 | 1 | 2 | 3 | | 4 | | 5 | | 6 | | 7 | | | 8 | | 9 | 10 |

How would you rate the fit around your forefoot?

| Loose | | |  | Perfect | | |  | |  | Tight | | |
| --- | --- | --- | --- | --- | --- | --- | --- | --- | --- | --- | --- | --- |
| 0 | 1 | 2 | 3 | 4 | 5 | 6 | 7 | 8 | | | 9 | 10 |

How would you rate the fit around your midfoot?

| Loose | | |  | Perfect | | |  | |  | Tight | | |
| --- | --- | --- | --- | --- | --- | --- | --- | --- | --- | --- | --- | --- |
| 0 | 1 | 2 | 3 | 4 | 5 | 6 | 7 | 8 | | | 9 | 10 |

How would you rate the fit around your heel?

| Loose | | |  | Perfect | | |  | |  | Tight | | |
| --- | --- | --- | --- | --- | --- | --- | --- | --- | --- | --- | --- | --- |
| 0 | 1 | 2 | 3 | 4 | 5 | 6 | 7 | 8 | | | 9 | 10 |

Is there anything that you liked about this shoe?

_____________________________________________________________________________________

Is there anything that you disliked about this shoe?

_____________________________________________________________________________________

**Please circle** any areas on the foot where you felt discomfort while running.


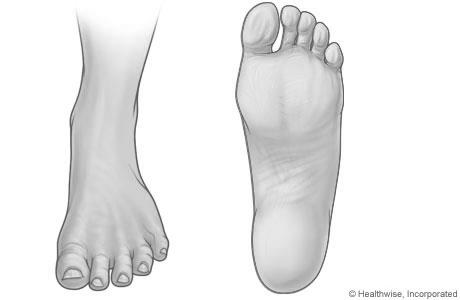

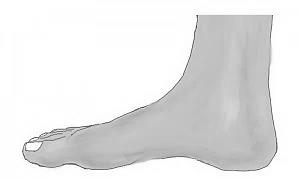

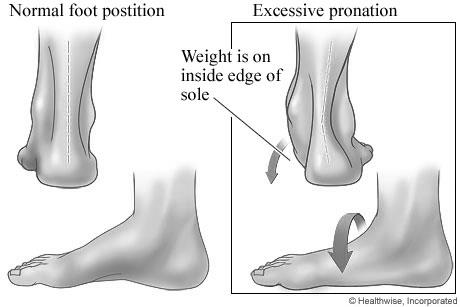


Shoe A2.

How do you feel this shoe performed running uphill?

| Poor |  |  |  |  |  |  |  |  |  | Great |
| --- | --- | --- | --- | --- | --- | --- | --- | --- | --- | --- |
| 0 | 1 | 2 | 3 | 4 | 5 | 6 | 7 | 8 | 9 | 10 |

How do you feel this shoe performed running level ground?

| Poor |  |  |  |  |  |  |  |  |  | Great |
| --- | --- | --- | --- | --- | --- | --- | --- | --- | --- | --- |
| 0 | 1 | 2 | 3 | 4 | 5 | 6 | 7 | 8 | 9 | 10 |

How do you feel this shoe performed running downhill?

| Poor |  |  |  |  |  |  |  |  |  | Great |
| --- | --- | --- | --- | --- | --- | --- | --- | --- | --- | --- |
| 0 | 1 | 2 | 3 | 4 | 5 | 6 | 7 | 8 | 9 | 10 |

What was your confidence level while running in this shoe (e.g. running downhill, technical terrain)?

| Timid | |  |  |  |  |  |  |  | Confident | |
| --- | --- | --- | --- | --- | --- | --- | --- | --- | --- | --- |
| 0 | 1 | 2 | 3 | 4 | 5 | 6 | 7 | 8 | 9 | 10 |

How was the overall fit of the shoe?

| Uncomfortable | | | |  | |  | |  | |  | |  |  | | Comfortable | | |
| --- | --- | --- | --- | --- | --- | --- | --- | --- | --- | --- | --- | --- | --- | --- | --- | --- | --- |
| 0 | 1 | 2 | 3 | | 4 | | 5 | | 6 | | 7 | | | 8 | | 9 | 10 |

How would you rate the fit around your forefoot?

| Loose | | |  | Perfect | | |  | |  | Tight | | |
| --- | --- | --- | --- | --- | --- | --- | --- | --- | --- | --- | --- | --- |
| 0 | 1 | 2 | 3 | 4 | 5 | 6 | 7 | 8 | | | 9 | 10 |

How would you rate the fit around your midfoot?

| Loose | | |  | Perfect | | |  | |  | Tight | | |
| --- | --- | --- | --- | --- | --- | --- | --- | --- | --- | --- | --- | --- |
| 0 | 1 | 2 | 3 | 4 | 5 | 6 | 7 | 8 | | | 9 | 10 |

How would you rate the fit around your heel?

| Loose | | |  | Perfect | | |  | |  | Tight | | |
| --- | --- | --- | --- | --- | --- | --- | --- | --- | --- | --- | --- | --- |
| 0 | 1 | 2 | 3 | 4 | 5 | 6 | 7 | 8 | | | 9 | 10 |

Is there anything that you liked about this shoe?

_____________________________________________________________________________________

Is there anything that you disliked about this shoe?

_____________________________________________________________________________________

**Please circle** any areas on the foot where you felt discomfort while running.


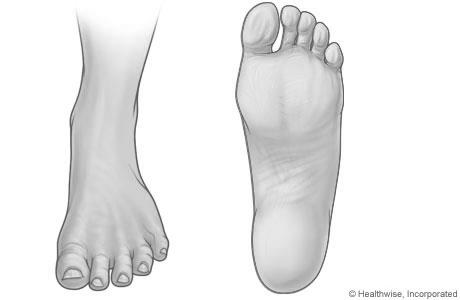

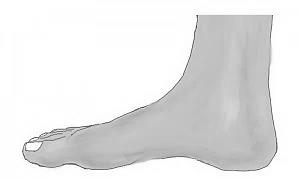

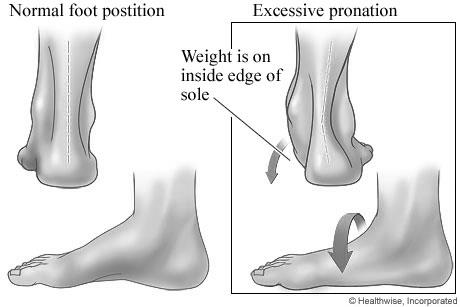

Supplement: Supplementary file 1 [file Datasheet1.docx]
